# Effects of core stability and feedback music on upper body mediolateral movements during cycling

**DOI:** 10.1186/s13102-024-00822-8

**Published:** 2024-03-21

**Authors:** Siwoo Jeong, Si-hyun Kim, Kyue-nam Park

**Affiliations:** 1 Department of Sports Rehabilitation Medicine, College of Smart Sports, Kyungil University, Gyeongsan, South Korea; 2 Department of Physical Therapy, Sangji University, Wonju, South Korea; 3 Department of Physical Education, Yonsei University, Seoul, South Korea

**Keywords:** Asymmetry, Auditory feedback, Core stability, Cycling, Head motion, Wearable sensor, Wireless earbud

## Abstract

**Background:**

Asymmetry in involuntary trunk motion during voluntary movements of the lower extremities is a risk factor for musculoskeletal injuries and may be related to core stability. Core stability plays a pivotal role in maintaining postural stability during distal segment movements. Because mediolateral head motion partially represents trunk motion during rhythmic movements, controlling it can help ensure symmetric trunk motion. This study aimed to investigate the relationship between core stability and asymmetric trunk motion during rhythmic movements, and to evaluate the effects of feedback music on mediolateral head motion.

**Methods:**

We developed a system that uses a wireless earbud and a high-resolution inertial measurement unit sensor to measure head angle and provide feedback music. When the head angle exceeds a predefined threshold, the music is muted in the earbud on the side of the head tilt. In our lab-based study, we measured head angles during cycling at 70% of maximum speed using this self-developed system, and compared them between individuals with good (Sahrmann core stability test: 2–5 level) and poor core stability (0–1 level). The amplitude of mediolateral head motion was represented by the difference between the left and right peak angles, and the symmetry in mediolateral head motion was represented by the average of left and right peak angles.

**Results:**

Individuals with poor core stability demonstrated significantly greater amplitude of, and less symmetry in, mediolateral head motion than those with good core stability. Additionally, feedback music significantly reduced the amplitude of mediolateral head motion in both the good- and poor-core-stability groups.

**Conclusion:**

Our findings indicate that core stability is crucial for maintaining symmetric head motion during rhythmic movements like cycling. Feedback music could serve as an effective tool for promoting symmetry in head motion and thus preventing musculoskeletal injuries.

## Background

Kinematic asymmetry increases the risk for musculoskeletal injury [1–4]. Musculoskeletal pain alters movement patterns, resulting in asymmetric trunk motion [2, 4, 5]. Asymmetrical trunk motion caused by asymmetrical pelvic pattern while sitting and standing is observed in people with lower back pain [2, 4]. People with knee osteoarthritis also show asymmetric lateral trunk sway during walking [1]. In addition, patellofemoral pain syndrome leads to excessive lateral trunk motion during stepping down and squatting [5, 6]. Symmetry of trunk motion during distal segment movements can be used as an indicator of abnormal musculoskeletal functioning.

Trunk motion during rhythmic activities such as walking or cycling is intricately connected with the body’s automatic balance maintenance mechanisms. This connection arises due to the inherent instability of the human body’s center of mass, subject to perturbations from body movements [7–9]. A critical component of this balance maintenance system is the anticipatory postural adjustments (APAs), essential for minimizing postural sway before a predictable disturbance, thereby contributing to fall prevention [9–11]. However, when APAs become dysfunctional, there is an excessive response of the trunk to voluntary limb movements. This phenomenon is observable during rapid arm raising, where the movement triggers a compensatory trunk movement in the opposite direction to maintain balance [12, 13]. The interplay between voluntary limb movements and trunk stability is an integral part of understanding core stability dynamics, particularly in activities like cycling.

APAs activate postural muscles prior to voluntary limb movements [14] and control the center of mass with respect to the center of pressure [15–18]. They provide core stability as feedforward control where the activity of deep muscles around the lumbopelvic segment (such as the transverse abdominis and diaphragm muscles) occurs prior to movements of the distal extremities [19–21]. A loss of the ability to control core stability alters trunk and pelvic motion patterns, which might result in musculoskeletal injury. A previous study demonstrated that cyclists with lower-back pain have altered trunk motion with loss of co-contraction of core muscles [22]. Dysfunction in the control of core muscles is associated with weak core stability, which causes APA dysfunction and asymmetric body motion during movements of the distal extremities. However, little is known about the relationship between core stability and asymmetric movements.

Morrison et al. reported that mediolateral head motion is associated with mediolateral trunk motion during gait, and restricting either motion affects the other [23]. Although the head and eyes are stabilized to maintain a stable gaze, head motion is partly affected by trunk motion during rhythmic movements such as walking, cycling, or rowing [24]. Therefore, symmetry in trunk motion can be evaluated based on mediolateral head motion.

**Fig. 1:**
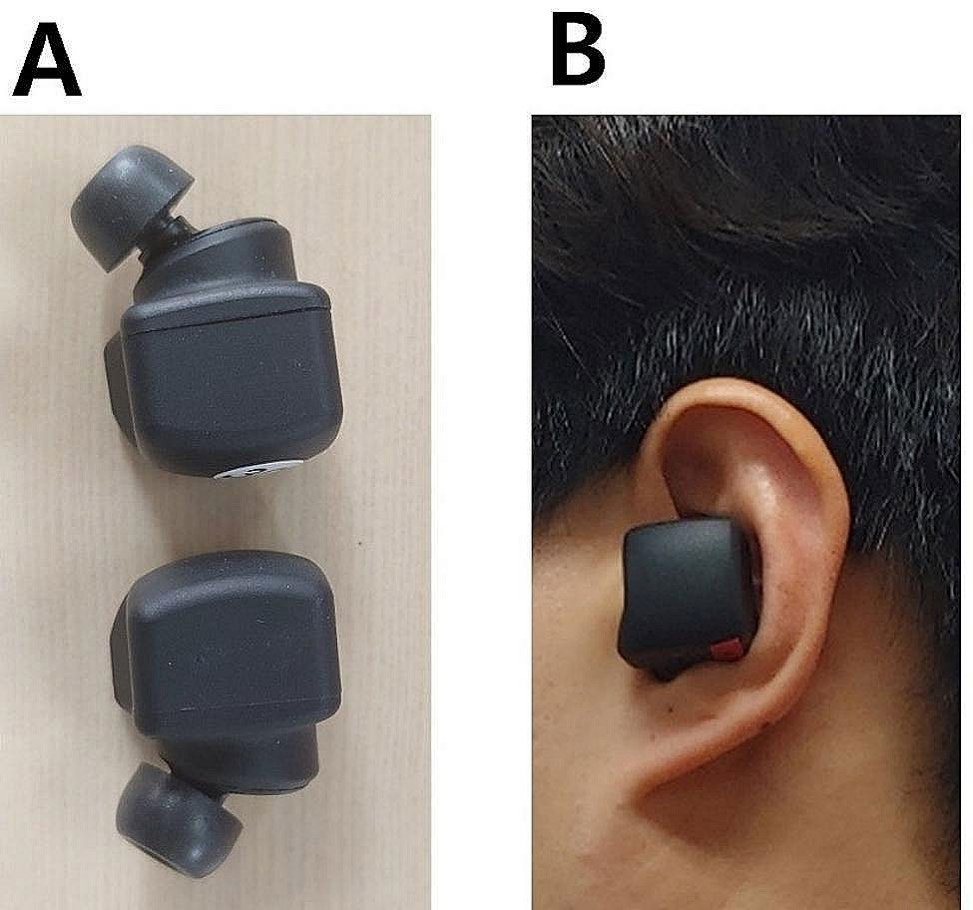
Wireless earbud and inertial measurement unit sensor (**A**). Wireless earbud worn in the ear (**B**)

**Fig. 2:**
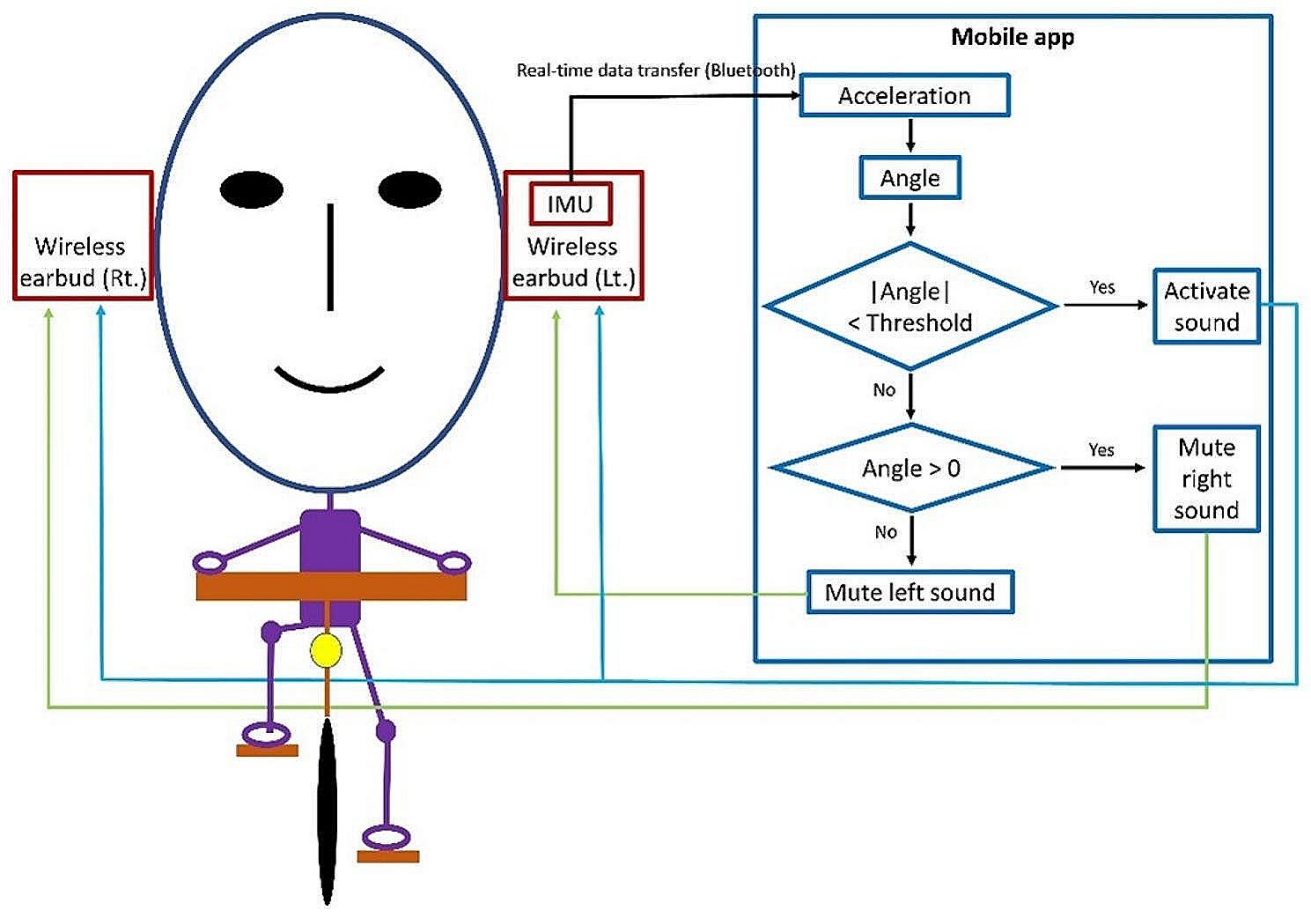
Schematic diagram of the self-developed system for measuring head angles and providing feedback music. A high-resolution inertial measurement unit (IMU) sensor was embedded in a left-side wireless earbud. IMU output was transferred to a mobile app in real time via Bluetooth. The mobile app calculated the head angle based on the accelerations provided by the IMU output. When the head angle was less than the predefined threshold, participants could listen to music from both earbuds. Otherwise, the wireless earbud on the side of the head tilt was muted

We developed a system that measures head angle and provides corresponding feedback based on the premise that head motion is associated with trunk motion. The system consists of a high-resolution inertial measurement unit (IMU) sensor embedded in a wireless earbud (Fig. 1). It uses the IMU output to determine the head angle. When the head angle exceeds a predefined threshold, the system mutes the earbud on the side of the head tilt. To listen to music in both ears, a person must maintain a head angle within the predefined threshold (Fig. 2). The system provides proprioceptive feedback based on head motion. Proprioceptive feedback on asymmetric motion produced by poor motor control can aid in reducing asymmetry.

We investigated the effects of core stability and feedback music on mediolateral head motion during cycling. Compared to walking, cycling involves relative restriction of movements of the upper and lower extremities and pelvis, which allows for more pronounced trunk and head movements. We hypothesized that a poor-core-stability group would have greater amplitude of and less symmetry in mediolateral head motion compared to a good-core-stability group. In addition, feedback music might improve symmetry in and reduce the amplitude of mediolateral head motion.

## Methods

This study enrolled 71 healthy participants (29 males and 42 females; mean age: 22.3 ± 2.2 years; mean weight: 166.6 ± 8.8 kg) who did not experience pain while cycling. The two independent variables in this design were core stability and feedback music, and the dependent variables were amplitude (*amp*) and symmetry index (*SI*) of mediolateral head motion during cycling. Participants were assessed for core stability using the Sahrmann Core Stability Test (SCST), following which they were divided into two groups - ‘good core stability’ and ‘poor core stability’. The participants then cycled under two conditions: with and without feedback music. The effects of core stability and feedback music on amp and SI were then statistically analyzed.

### Participants

Participants were recruited through a combination of online advertisements and flyers posted around the University campus and its vicinity. Interested individuals contacted the study team via the contact details provided on the advertisements. Prior to enrollment, potential participants were screened for eligibility based on the following inclusion criteria: (1) healthy individuals aged 18 and above, (2) individuals able to perform cycling without experiencing any pain, and (3) individuals who did not meet any of the exclusion criteria. Exclusion criteria involved pregnancy and the presence of vestibular, neurological, cardiopulmonary, psychological, or musculoskeletal disorders. To assess whether participants experienced pain during cycling, we asked them directly with the question: “Do you experience any pain while cycling?” If a participant responded affirmatively, they were excluded from the study. This method allowed us to ensure that all included participants were comfortable performing the cycling tasks required for our study. A total of 71 healthy participants (29 males and 42 females; mean age: 22.3 ± 2.2 years; mean weight: 166.6 ± 8.8 kg) were successfully recruited for the study. All participants in the study were amateur cyclists, meaning they knew how to ride a bicycle but did not cycle on a regular basis. This study did not specifically aim to measure cycling proficiency or frequency of cycling; however, all participants were capable of carrying out the cycling tasks required for our research. Their level of cycling experience can be broadly defined as beginner or casual, rather than intermediate, advanced, or professional. The study was in accordance with the principles as outlined in the Declaration of Helsinki. The participants provided written informed consent. The study was approved by the institutional review board of the Jeonju University.

### Sahrmann core stability test (SCST)

**Fig. 3:**
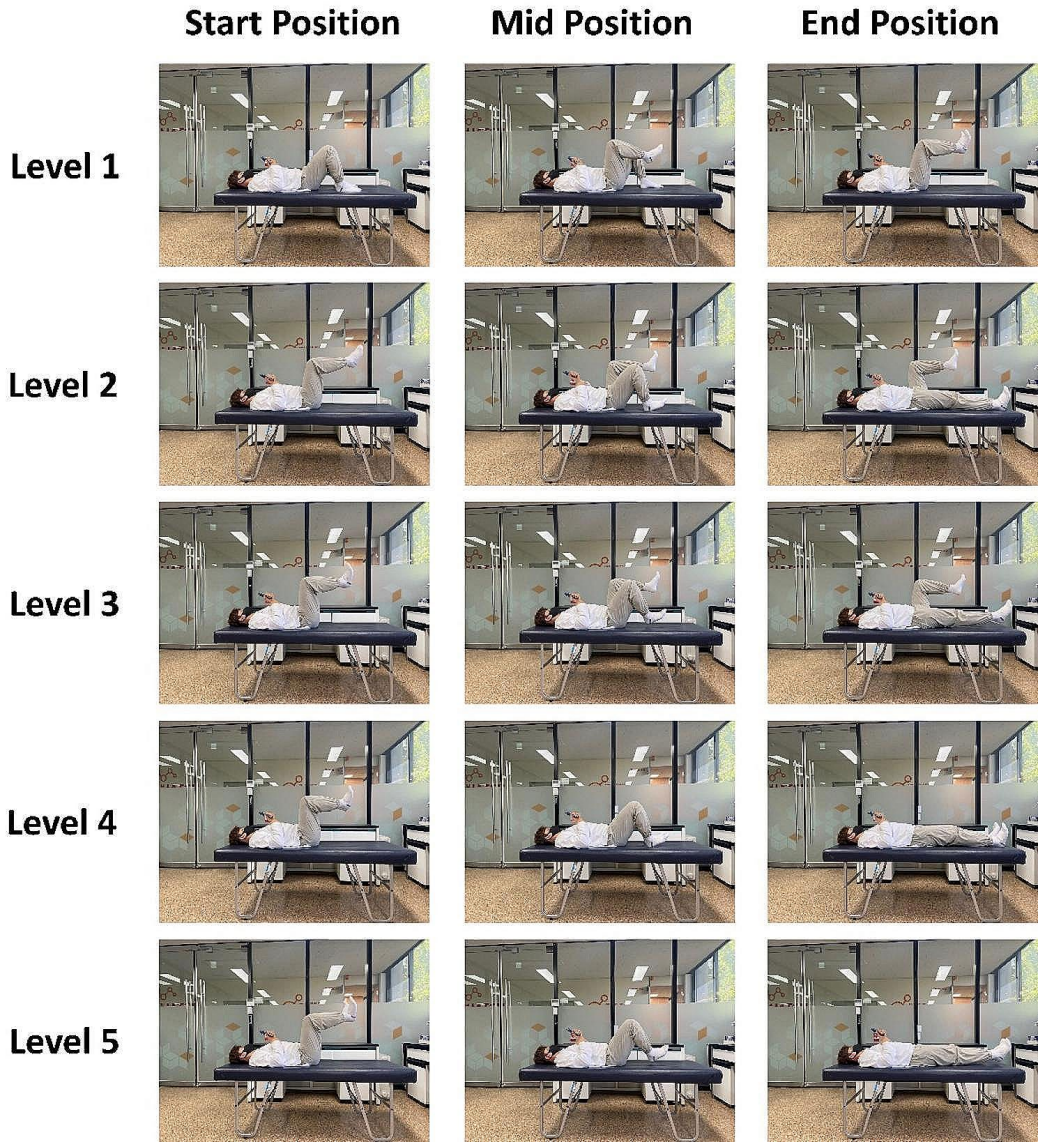
The five levels of the Sahrmann core stability test

The SCST was performed to evaluate core stability. It included five progressively more difficult tasks. The inflatable pad of a stabilizer pressure biofeedback unit (Chattanooga Group, Hixson, TN, USA) was placed in a natural lordotic curve while participants were placed in a crooked lying position. The pad was inflated to 40 mmHg before the task. A deviation of > 10 mmHg during the task indicated loss of stabilization of the lumbopelvic hip complex by the stabilizer muscles. Participants who completed a task without a deviation of > 10 mmHg were instructed to perform the next task. Performance (i.e., the ability to complete the tasks without a deviation of > 10 mmHg) was rated on a 5-point scale (Fig. 3). Participants were divided into two groups based on their SCST scores: ‘poor core stability’ (0–1) and ‘good core stability’ (2–5). This categorization was adopted from a previous study where an SCST of 1 or lower was deemed indicative of poor core stability [25]. The tasks were performed as reported previously [26].

### Instruments

A high-resolution single IMU (BNO080; Ceva Technologies, Rockville, MD, USA) equipped with a triaxial accelerometer and triaxial gyroscope was embedded into a left-side wireless earbud (QCY-T1C; Dongguan Hele Electronics, Dongguan, China) to measure head angle (Fig. 1). IMU data were collected at 100 Hz. Each sample contained signed 16-bit acceleration output for x, y, and z axes. The acceleration outputs were transferred to a self-developed mobile app (DDoARi, Republic of Korea) via Bluetooth. The app calculated the mediolateral head angle in real time, similar to a previous study [27], and controlled the volume of music from the wireless earbud to provide feedback.

### Feedback music

Feedback music was provided in real time to prevent excessive mediolateral head motion during cycling. If the mediolateral head angle exceeded a predefined threshold, the wireless earbud on the side of the head tilt was muted. Once the mediolateral angle returned to the set range, the muted earbud was unmuted (Fig. 2). For example, if the angle threshold was 10° and the head tilted > 10° to the right side, the earbud on the right side was muted; the muted earbud was unmuted when the mediolateral head angle was reduced to < 10°.

### Cycling

Participants wore the wireless earbud, including the IMU sensor, in their ears to measure head angle in the frontal plane and receive feedback music. The cycling was performed on an indoor cycle (Iwha Sean Lee X ike Inc., Republic of Korea). The cycling speed was measured and monitored in real-time by a device installed on the cycle, and the speed was displayed on an installed monitor, which allows participants to maintain their target speed. The investigator supervised the experiment to ensure that the participants did not deviate from the target speed and provided continuous guidance to help them maintain the target speed. During warm-up, participants cycled for 5 min at their preferred speed. Then, after a 5 min rest period, the participants were instructed to cycle at the fastest speed possible; 70% of the measured maximum speed was set as the target speed.

After cycling at their fastest speed possible, participants were provided a 5-minute rest period to prevent undue fatigue. Following this rest period, participants engaged in trials at 70% of their measured maximum speed with and without feedback music. In the trial with no feedback, participants were asked to cycle at the target speed for 1 min. The mediolateral head angle was measured for 1 min during cycling at the target speed, and data from the final 40 s were analyzed. We chose to analyze the final 40 s of data from each trial for a couple of reasons. Firstly, as participants were trying to reach their set velocity, the time taken to achieve that speed varied between individuals. By focusing on the final 40 s, we ensured that we were analyzing data collected when participants were likely cycling at their target velocity. A value of 50% of the measured maximum mediolateral head angle was set as the threshold for feedback music, which was a medium tempo piece (125 beats per minute) in a minor key. The exact music piece used for feedback can be found at the following link: https://music.bugs.co.kr/album/20500357?wl_ref=list_ab_01_ar. After the trial with no feedback, participants cycled for 1 min at the target speed and received feedback. The participants rested for 3 min between the trials with and without feedback.

### Symmetry

**Fig. 4:**
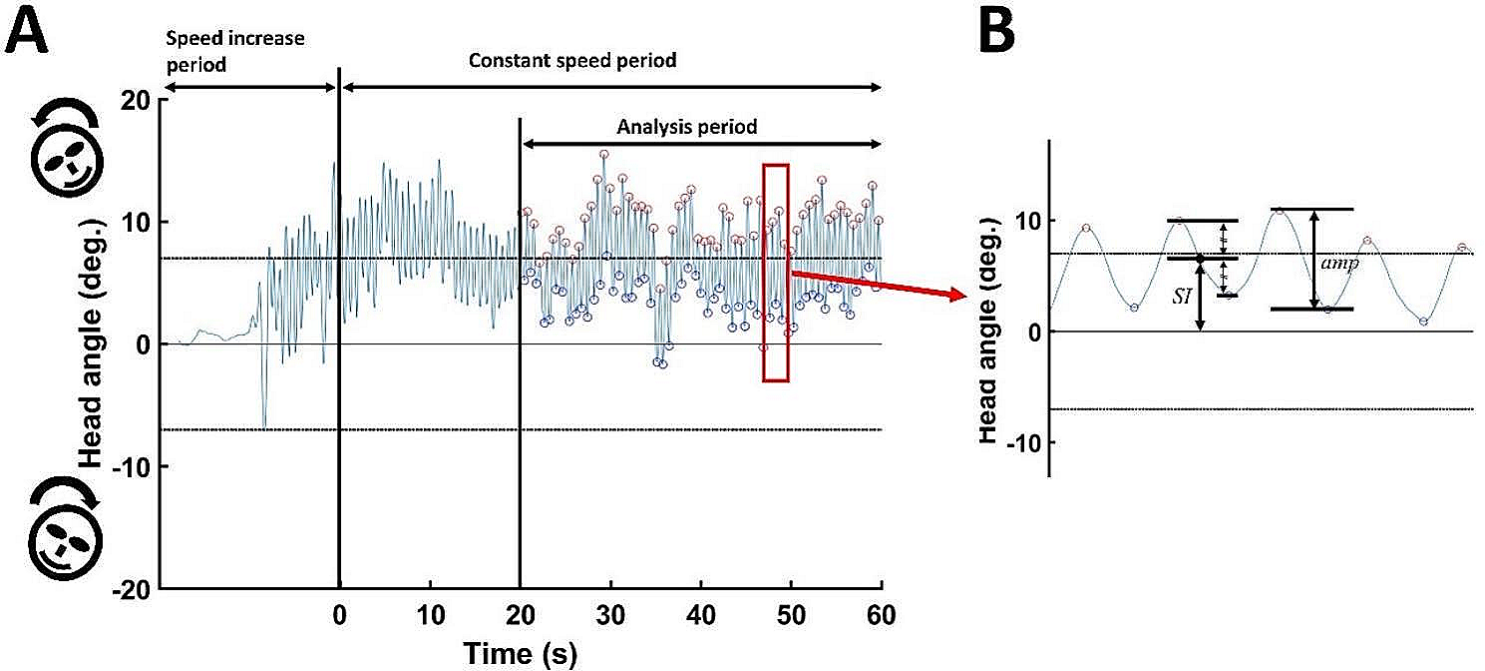
Mediolateral head angle (**A**) and its symmetry and amplitude (**B**). (**A**) After approaching 70% of the measured maximum speed, participants cycled for 1 min at a constant speed. The sign of the angle represents the direction of head tilt. Positive and negative angles indicate right and left directions, respectively. Data for the final 40 s after the participant reached the target speed were analyzed. The right-side (**A**, red circles) and left-side (A, blue circles) peak angles for each cycle were used to calculate symmetry (*SI*) in and the amplitude (*amp*) of the head angle. (**B**) The average right and left peak angles demonstrate symmetry in mediolateral head movement. An *SI* value of 0 indicates perfectly symmetric head movement. The amplitude of mediolateral head movement was calculated as the difference between the right and left peak values

The maximal right and left head angles during each cycle were used to evaluate the range of mediolateral head motion and symmetry in head angle during cycling. Positive and negative signs for head angle represent the right and left directions, respectively. Therefore, the maximal value was the maximal right head angle, and the minimal value was the maximal left head angle (Fig. 4A).

The maximum and minimum values during each cycling cycle were recorded. The average difference between the maximum and minimum values indicated the range of mediolateral head motion:

$$ amp=\frac{\sum _{i}({max}_{i}-{min}_{i})}{n}$$

Here *n* is the number of peak values and *max* and *min* represent the maximum and minimum values during each cycling cycle, respectively (Fig. 4B).

Head angle symmetry during cycling was represented by the average of the maximal and minimum values:

$$ SI= \frac{\sum _{i}\left|({max}_{i}+{min}_{i})/2\right|}{n}$$

For a perfectly symmetric mediolateral head angle, the maximal right and left head angles should be equal (i.e., symmetry index [*SI*] = 0). Because the values for each direction have opposite signs, the closer the *SI* value is to 0, the more symmetric the head angle is in the frontal plane (Fig. 4B).

### Statistical analyses

Independent variables included core stability and feedback music, while the dependent variables were *amp* and *SI*. A 2 × 2 mixed-model analysis of variance (ANOVA) was employed to assess the effects of core stability and feedback music on *amp*. Another 2 × 2 mixed-model ANOVA was used to evaluate the core stability and feedback music on *SI*, with a significance level set at *p* < 0.05. The partial eta squared value (*η*_p_^2^) was calculated to describe the effect size.

## Results

Of the 71 participants, 25 and 46 were included in the poor and good-core-stability groups, respectively (SCST scores of 0–1 and > 1, respectively). There were no significant differences between the groups in terms of age, height, weight, body mass index, or maximal cycling speed. The visual analog scale scores for pain were significantly higher in the poor-core-stability group compared to the good-core-stability group (Table 1); however, participants in both groups successfully cycled for more than 1 min without pain.

**Table 1:** Comparison of demographic characteristics between the poor- and good-core-stability groups

	Poor	Good	*p* value
Males/ Females (n)	13/12	16/30	-
Age (years)	22.0 ± 2.8	22.3 ± 1.8	0.53
Height (cm)	167.1 ± 8.8	166.3 ± 8.9	0.72
Weight (kg)	64.8 ± 12.4	61.7 ± 13.6	0.35
Body mass index (kg/m^2^)	23.1 ± 3.2	22.3 ± 3.1	0.35
VAS pain score (cm)	4.6 ± 2.6	3.0 ± 3.2	0.04
Max cycling speed (km/h)	35.7 ± 8.2	32.9 ± 6.2	0.10

VAS: visual analog scale

**Fig. 5:**
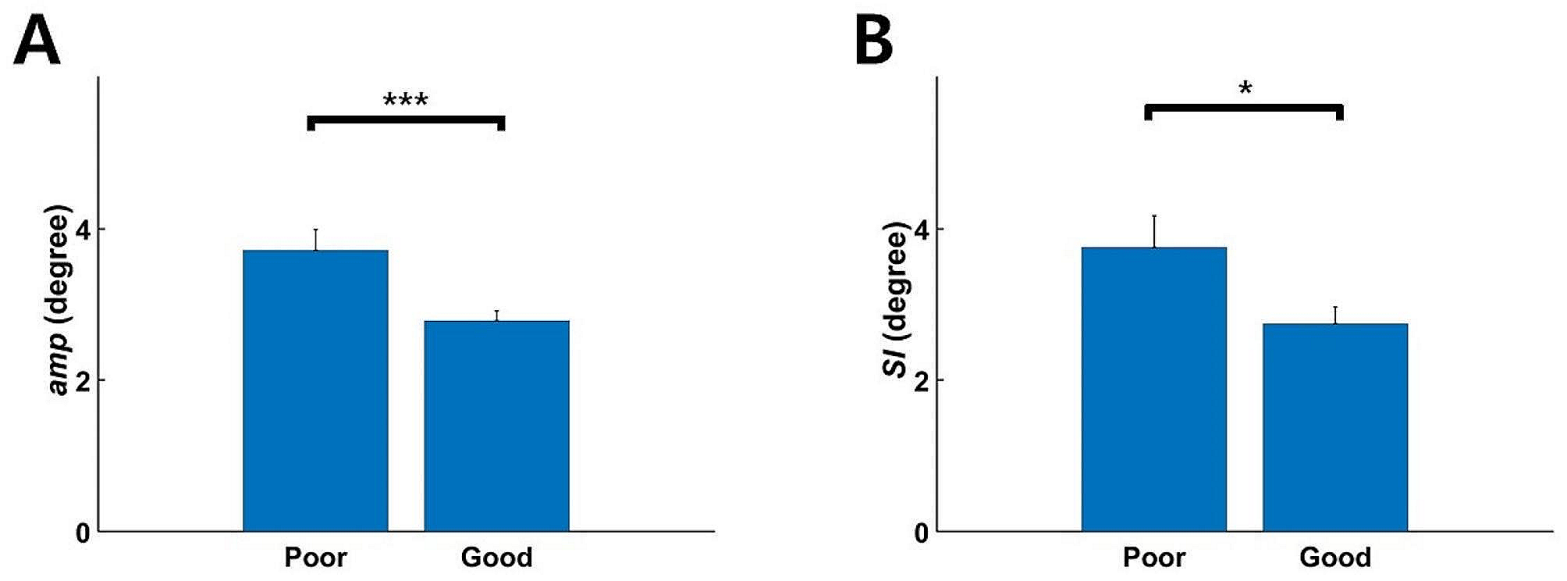
Mean (± SE) amplitude of (**A**) and symmetry (**B**) in mediolateral head motion in the poor- and good-core-stability groups. (**A**) The amplitude of mediolateral head motion was significantly higher in the poor-core-stability group compared to the good-core-stability group during cycling (ANOVA, ^***^p < 0.001). (**B**) An *SI* of 0° indicates symmetric mediolateral head motion. The poor-core-stability group demonstrated significantly less symmetry in mediolateral head motion during cycling compared to the good-core-stability group (ANOVA, ^*^p < 0.05)

**Fig. 6:**
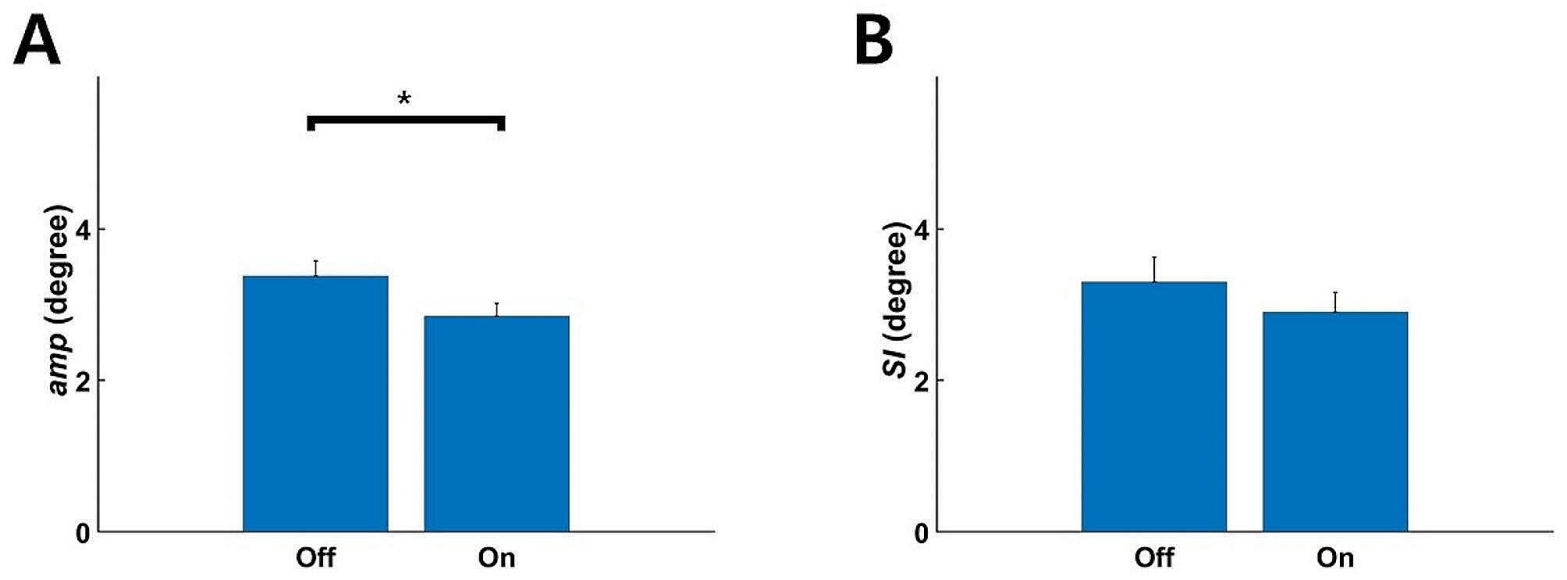
Effect of music feedback on mean (± SE) amplitude of (**A**) and symmetry in (**B**) mediolateral head motion. (**A**) The feedback music significantly reduced the amplitude of mediolateral head motion during cycling (ANOVA, ^*^p < 0.05). (**B**) However, it did not significantly improve symmetry in mediolateral head motion during cycling (ANOVA, *p* = 0.16)

The ANOVA demonstrated significant effects of core stability (*p* < 0.001, *η*_p_^2^ = 0.083) and feedback music (*p* < 0.05, *η*_p_^2^ = 0.031) on movement amplitude (*amp*); however, the interactive effect of core stability and feedback music on *amp* was not significant (Table 2; Fig. 5). The effect of core stability (ANOVA, *p* < 0.05, *η*_p_^2^ = 0.039) on the symmetry index (*SI*) was significant, but those of feedback music and the interaction were not significant (Table 3; Fig. 6).

**Table 2:** Two-way analysis of variance of the effects of core stability (good vs. poor) and feedback music on the amplitude of mediolateral head motion

	df	Sum Sq	Mean Sq	F value	*p* value	η_p_^2^
Core stability	1	28.0	28.0	12.4	< 0.001	0.083
Feedback music	1	9.8	9.8	4.3	< 0.05	0.031
Interaction	1	0.12	0.12	0.05	0.82	< 0.001
Error	138	310.6	2.3			
Total	141	348.7				

**Table 3:** Two-way analysis of variance of the effects of core stability (good vs. poor) and feedback music on symmetry in mediolateral head motion

	df	Sum Sq	Mean Sq	F value	*p* value	η_p_^2^
Core stability	1	32.8	32.8	5.6	< 0.05	0.039
Feedback music	1	11.8	11.8	2	0.16	0.014
Interaction	1	15.9	15.9	2.7	0.10	0.019
Error	138	815.1	5.9			
Total	141	869.4				

## Discussion

We investigated the effects of core stability and feedback music on mediolateral head motion during cycling. The amplitude of, and symmetry in, head angles in the frontal plane were used to evaluate mediolateral head motion during cycling. We found that mediolateral head motion during cycling was associated with core stability and that the amplitude of the motion was successfully controlled with feedback music, but not the symmetry in the motion.

The amplitude of mediolateral head motion during cycling was greater in the poor-core-stability group compared to the good-core-stability group (Fig. 5A). Because indoor cycling partly restricts the motion of lower extremities in the frontal plane as well as the motion of upper extremities, the reaction force produced by pedaling is transferred to the upper body with minimal additional compensatory motion of the lower and upper extremities. Core stability stabilizes the proximal body to allow distal movements [28]. Deep muscles, including the multifidus and transverse abdominis (i.e., the core muscles), are activated prior to movements of the lower extremities [19–21]. Altered control of the core muscles leads to weak proximal stability, which causes increased trunk and head motion due to the reaction force.

In our study, the poor-core-stability group had greater asymmetry in mediolateral head motion during cycling compared to the good-core-stability group (Fig. 5B). Poor core stability is characterized by dysfunctional control of the core muscles, bilaterally asymmetric activity of the core muscles, and consequently asymmetric movement. Asymmetry causes pain because of bilateral imbalance produced by suboptimal spine stability [29]. Altered spinal alignment is a mechanical cause of lower-back pain [30]. Cyclists with nonspecific lower-back pain demonstrate altered kinematics of the lower lumbar spine due to the loss of appropriate motor control of the lower lumbar multifidus [22]. Taken together, our findings imply an interaction among core stability, symmetric movement, and pain. Poor core stability induces asymmetric movement, and repetitive asymmetric movement causes musculoskeletal pain or injury.

The SCST measures the ability to control the lumbopelvic segment [26, 31, 32]. Participants are instructed to draw in the abdominis, maintain the lordotic curve, and lower the legs. Our findings imply that the SCST evaluates the preprogrammed feedforward core muscle contraction required to maintain proximal stability. In this study, head and trunk motions during cycling were partly controlled by the feedforward activity of the core muscles. Because the participants were instructed only to maintain the target speed during cycling with no mention of postural stability, they did not intentionally control their upper-body motion. The mediolateral head motion of the poor-core-stability group during cycling was greater than that of the good-core-stability group. Therefore, the ability to control the lumbopelvic segment voluntarily could reflect involuntary postural stability against the reaction force generated by distal segment movements.

Feedback music successfully controlled excessive mediolateral head motion during cycling. The feedback informed the participants of the direction of head tilt by removing the sound being produced by the corresponding earbud. Because mediolateral head motion occurred periodically during cycling, the earbuds were muted only briefly. Feedback music significantly reduced the amplitude of mediolateral head motion in both the poor- and good-core-stability groups with no decrease in cycling speed (Fig. 6A), however did not improve the symmetry of motion (Fig. 6B). Although the earbud was muted only briefly, it was still effective for informing the participants that their head was excessively tilted.

While the music feedback effectively reduced the amplitude of mediolateral head motion, it did not significantly improve symmetry in the head motion. This disparity may be attributed to different aspects of movement dynamics and distinct biomechanical factors. The amplitude of motion is largely governed by the magnitude of force exerted by muscles, which can be consciously modulated based on external cues such as our music feedback. On the other hand, symmetry in motion involves a complex interplay of bilateral muscle coordination, proprioception, and biomechanical factors such as skeletal alignment and joint stability. These factors may be less amendable to rapid adjustment through auditory feedback alone. Furthermore, the effect of music feedback on motion symmetry could be confounded by pre-existing asymmetries, which might require a more sustained and targeted intervention to address. Further research will investigate these possibilities and to develop interventions aimed at improving symmetry in motion during physical activities such as cycling.

In this study, it was assumed that involuntary mediolateral head motion represents involuntary whole-upper-body motion produced by distal segment movements during cycling. However, how mediolateral head motions are controlled in response to feedback music is unknown. If head motions are controlled by improved postural stability, controlling mediolateral head motion would reduce the amplitude of mediolateral trunk motion. Otherwise, only neck muscles would be more controlled to prevent the excessive mediolateral head motion. If trunk motions contribute to head motion, cycling with feedback music could be used as part of training programs to improve core stability, since it improves the ability to stabilize the lumbopelvic segment against movements of the lower extremities. Further research is needed to determine how participants modify mediolateral head motion in response to feedback music.

One significant strength of our study is its novel use of feedback music as a method to control the amplitude of mediolateral head motion during cycling. This method demonstrated efficacy in reducing the amplitude in both poor- and good-core-stability groups, suggesting a potential novel approach to training programs aimed at improving core stability. Another strength is our approach to measuring core stability. By leveraging the technology in wireless earbuds and an IMU sensor, we were able to assess core stability without the need for a trained professional, indicating a more practical and accessible means of evaluating this crucial element of physical health and performance. However, our study also has several limitations. One limitation is that our measure of involuntary mediolateral head motion may not capture all relevant dynamics of upper-body movement during cycling. Specifically, we did not directly measure trunk motion, which might play a significant role in the kinematics produced by voluntary movements of the lower limbs. Secondly, we did not evaluate the correlation between involuntary head motion and trunk motion in detail. This correlation might be important for a fuller understanding of how feedback music influences movement dynamics during cycling. Finally, our assessment of participants’ cycling experience and ability was relatively basic. Given that cycling ability and experience may influence core stability and mediolateral head motion, more nuanced measurement of these variables could provide further insights. Given these limitations, future research is necessary to expand on our findings and to further clarify the relationship between involuntary head and trunk motions. In particular, it will be important to investigate whether feedback music or other forms of sensory feedback could be used to improve symmetry in motion, a dimension of movement dynamics that our feedback music intervention did not significantly affect.

## Conclusions

The SCST results showed that mediolateral head motion during cycling had greater amplitude and greater asymmetry in the poor-core-stability group compared to the good-core-stability group. The ability to control movements of the lumbopelvic segment indicates postural stability against movements of the distal extremities. In addition, feedback music reduced the amplitude of mediolateral head motion. Indoor cycling with feedback music can be incorporated into training programs to improve core stability.

## Data Availability

The data used in the current study are available from the corresponding author upon reasonable request.
